# Consumer motivation for organic food consumption: Health consciousness or herd mentality

**DOI:** 10.3389/fpubh.2022.1042535

**Published:** 2023-01-11

**Authors:** Jiangbo Wang, Yefei Xue, Tingting Liu

**Affiliations:** ^1^School of Economics, Shenzhen Polytechnic, Shenzhen, China; ^2^Business School, Nanjing University, Nanjing, China; ^3^Shenzhen Center for Disease Control and Prevention, Shenzhen, China

**Keywords:** consumer motivation, organic food consumption, propensity score matching, health consciousness, herd mentality

## Abstract

Whether health awareness is self-awareness or conformity awareness is a question worth discussing. Especially under the current trend of organic food consumption, whether it is driven by health consciousness or herd mentality is worth exploring. This is not only about the government's formulation of health policies for the industry (for example, paying more attention to health standards), but also about the sustainable development of organic food consumption (for example, suppliers highlighting their own health advantages). However, there is still little research in this area. Based on data from 1,658 respondents in four first-tier cities in China, this paper explores whether consumers are consuming organic food under health consciousness or herd mentality. This paper uses a logit model to explore the key causes of health consciousness or herd mentality, and uses a propensity score matching (PSM) method to measure the impact of health consciousness and herd mentality on organic food consumption, comparing the effects across age and education groups. The results show that: (1) Individual characteristics, family characteristics, health status, volition, social trust and market education significantly influenced consumers' health consciousness or herd mentality; (2) The main motivation for organic food consumption is health consciousness, but herd mentality plays a role of amplifying this effect.

## Introduction

Organic food, known as ecological or biological food, is a relatively unified international term for uncontaminated natural food. Organic food usually comes from organic agricultural production systems and is produced according to international organic agricultural production requirements and corresponding standards (1). In domestic and international literature, organic food is sometimes translated as organic product, but this paper does not distinguish between them, so organic product is equivalent to organic food.

In recent years, people are more concerned about their health than ever before. Public opinions on food safety have continued to emerge. Food safety has become one of the most realistic issues in society and of utmost concern to residents. In such an environment, people have taken the consumption of organic food as an alternative to avoid the food safety problems. According to the World of Organic Agriculture Statistics and Emerging Trends 2022 (WOASET 2022) published by the Swiss Research Institute of Organic Agriculture (FiBL) and IFOAM – Organics International, over 74.9 million hectares of organic agricultural land were recorded in 190 countries in 2020.

China is a country that has paid more importance to the public's health and food safety. The Health China Initiative (2019–2030) issued by the Chinese government advocates the need for a sensible and reasonable diet. The Chinese government has made efforts to improve the quality of agricultural products and food safety. According to the WOASET 2022, organic agricultural land of China was over 2.4 million hectares, ranking second in Asian, and organic food market reached 10.2 billion Euros.^1^ Organic vegetables are the typical examples. They are becoming increasingly popular among the public due to their unique safety and health characteristics. Now there has been a huge potential demand for organic vegetables in China.

However, there is a serious information asymmetry in the organic food market, for the variety of organic vegetables is vast, and the cost for consumers to fully understand these various organic food products is high (2). It is difficult for consumers to make rational consumption decisions, and they may adopt a follow-the-leader strategy under the influence from people around them. Clarifying the drivers of organic food consumption and identifying the key factors influencing consumers' consumption, are important to ensure stable growth in organic food demand, prevent price fluctuations and guide the high quality development of the industry (3). Therefore, which force is behind the high growth in organic food consumption in China: The awakening of health consciousness or the influence of herd contagion? And how do these two forces influence consumption? Answering these questions and exploring the mechanisms are the task of this paper.

First, let's discuss the literature of research on the development of organic food industry. The existing literature on the industrial development and market consumption of organic food can be broadly divided into two dimensions: macro level and micro level. At the macro level, scholars have focused on the exploration of industrial policies, supply and demand equilibrium, and price fluctuations. At the micro level, the focus is on the analysis of organic vegetable production behavior, mainly concerning about the production intentions, technical efficiency of production and key influencing factors (4). These studies revealed real-life problems on the supply side of organic food industry, such as poor resource endowment, sloppy cultivation management and high yield fluctuations, and gave policy recommendations tailored to local conditions, which serve as a guide for the development of organic food industry. However, microscopic research on the demand side, such as consumers' consumption of organic food, is relatively lacking (5). Although some studies in green product consumption areas had regarded organic food as a characterization indicator, but these studies contributed little to guiding the organic food industry (6, 7). In order to promote the high-quality development of the organic food industry, there is still need to explore the motivation mechanism of consumers' organic food purchasing behavior (8, 9).

Second, let's discuss the consumer behavior in organic food. At present, there is rich existing literature on consumer behavior, pathways of action and influencing factors. The studies have evolved from the founding of consumer behavior in the 1970's (10) to the theories of planned behavior (11) and the consumer culture (12), and to the currently prevalent views on irrational behavior (13). Based on general psychological characteristics of individuals and external factors, deep-seated explanations of consumers' internal perspective are analyzed to reveal consumer decision-making processes, including attitudes, preferences, relationships and choices. However, there is room for improvement in the existing empirical evidence: (1) In terms of research methodology, the existing literature mostly adopts Logit and Probit methods, incorporating herding behavior as a dummy variable into the equation and directly comparing the differences in individual rational and irrational behavior, and ignoring the heterogeneity between different groups. (2) In terms of research content, the existing literature focuses on a single discussion of the rational person hypothesis or the irrational hypothesis; human decision-making is influenced by the interaction of individual rationality and sociality, and needs to be considered in an integrated manner (12). In the studies on China, most of the existing literature uses primary research data from provinces, but the vast size of China makes it difficult to reflect geographical differences in the samples from the level of provinces.

In order to uncover the motivations for organic food consumption, this paper will explore the key triggers of health awareness and herding behavior based on data from 1,658 respondents in four first-tier cities in China: Beijing, Shanghai, Guangzhou and Shenzhen. This study will measure the effects of health awareness and herding behavior on organic food consumption using the propensity score matching (PSM) method. The study will also use primary data from field research, take into account the heterogeneity of different groups, and consider the interactive effects of human decisions. The findings of this paper will be useful in broadening the research on the effect of health concepts on organic food consumption. It can be helpful to the government's formulation of health policies for the industry and the sustainable development of organic food consumption.

## Economic explanations and research hypotheses

In order to clarify the influence of personal health awareness and herd mentality in organic food consumption, the information waterfall model constructed by Anderson and Holt (14) is used in this paper. It is assumed that residents have two choices of consuming and not consuming organic food, denoted as strategy Y and strategy N respectively, i.e., the strategy set R = {Y, N} and the actual action v ∈ R. The prior probabilities that residents consume organic food or not are set as P(Y) and P(N), and P(Y) + P(N) = 1, which represents the residents' initial consumption attitude. When consumers believe that consumption of organic food brings higher utility than similar products, we have P(Y) > P(N), and *vice versa* (15).

Let δ_*i*_ = (*y, n*), *i* = 1,2, …, *n* be the private information of resident *i* about the consumption of organic food, δ_*i*_ = *y* means that the utility of organic food is higher than that of similar products, δ_*i*_ = *n* means that the utility of consuming organic food is lower. Since private information is incomplete information about the decision outcome, we set *p*_*i*_ as the probability that resident *i*'s private information is consistent with the decision outcome (16). Then we have


$$P(v|\delta_{i}=y)=\left\{ \begin{array}{l} \;p_{i},\;v=y\\ 1-p_{i},\;v=n \end{array}\right.$$

$$P(v|\delta_{i}=n)=\left\{ \begin{array}{l} 1-p_{i},\;v=y\\ \;p_{i},\;v=n \end{array}\right.$$

Private information is consistent with the decision outcome while *p*_*i*_ takes values closer to 1, and private information is inconsistent with the decision outcome while *p*_*i*_ takes values closer to 0.5. It is assumed that the public information is published as a Bayesian rule (there is a sequential order), while the later decision maker can observe the information of the earlier decision maker and adjust their options (17). Before resident *i*, there are already K individuals choosing organic food consumption and M choosing alternatives, i.e., K + M = *i*-1. Then, the probability that resident i's judgment of the kth other resident's organic food consumption is accurate can be expressed as $p_{ik}^{{}^{\prime }}$, and the probability that the mth individual chooses not to consume organic food can be expressed as ${\;p}_{im}^{{}^{\prime }}$. Then $p_{ik}^{{}^{\prime }}$, ${\;p}_{im}^{{}^{\prime }}$ ∈ (0,1), *k* = 1,2,…, *K*; *m* = 1,2, …, *M*. Thus, the probability of consumer organic food consumption can be expressed as:


$$\begin{array}{l} P\left(Y|K,M,\delta_{i}=y\right)=\frac{P\left(K,M|Y\right)P\left(Y\right)p_{i}}{P\left(K,M|Y\right)P\left(A\right)p_{i}+P\left(K,M|N\right)P\left(N\right)\left(1-p_{i}\right)} \end{array}$$

$$\begin{array}{l} =\frac{1}{1+\frac{1}{\left(\prod_{k=1}^{K}\frac{p_{ik}^{{}^{\prime }}}{1-p_{ik}^{{}^{\prime }}}\times \prod_{m=1}^{m}\frac{1-p_{im}^{{}^{\prime }}}{p_{im}^{{}^{\prime }}}\times \frac{P\left(Y\right)}{1-P\left(Y\right)}\times \frac{p_{i}}{1-p_{i}}\right)}} \end{array}$$

Where $\frac{P\left(Y\right)}{1-P\left(Y\right)}$ is the ratio of organic food consumption group to non-organic food consumption group in the initial state (18), the size of which is determined by the environment in which the residents live; $\frac{p_{i}}{1-p_{i}}$ and $\frac{{1-p}_{i}}{p_{i}}$ are the ratios of organic food consumption to non-organic food consumption in the private information display, reflecting the health consciousness of individuals or not; $\prod_{k=1}^{K}\frac{p_{ik}^{{}^{\prime }}}{1-p_{ik}^{{}^{\prime }}}\times \prod_{m=1}^{m}\frac{1-p_{im}^{{}^{\prime }}}{p_{im}^{{}^{\prime }}}$ is the ratio of organic food consumption to non-organic food consumption for others in the public information display, reflecting the impact of group organic food consumption behavior, i.e., herd mentality. Thus, the probability of organic food consumption can be expressed as an interaction of environmental characteristics (19), personal health awareness and herding behavior: *P*(*Y*|*K, M*) ∝ *Environmental features* × *Personal Health Awareness* × *Herd mentality*.

In summary, this paper proposes the following hypotheses:

1. Personal health awareness has a significant positive effect on organic food consumption.
2. The herd mentality of the population has a significant positive impact on the consumption of organic food.
3. Environmental characteristics have a significant impact on organic food consumption.
4. Personal characteristics, family characteristics, regional characteristics, volitional attributes and health status have significant effects on herding mentality.
5. Individual characteristics, family characteristics, regional characteristics, quality of will, health status, etc. have significant influence on health awareness.

Then we focus on the interpretation of hypotheses 4 and 5.

First, we explain the hypotheses 4. Consumer beliefs indirectly influence consumption intentions and behavior through individual characteristics and socio-cultural factors (such as gender, age, education, income, experience) (20). Psychological research defines herding as an individual's adherence to the social norms of the group to which he or she belongs (21) and herding as a way for consumers to change their attitudes, product evaluations and purchase decisions by referring to the product evaluations or purchase behavior of the group (22). This is manifested in the decision to follow the behavior of others by considering their preferences as a predetermined solution (23). It is the intersection of the dimensions of under-information and following in terms of identification and measurement (24). The former is the belief that other people's preferred choices or opinions are often correct or better, and that information in a state of information ambiguity motivates herding behavior; the latter is the belief that other people's opinions or preferences are not necessarily better, and that herding is a way of being socially accepted. The latter believe that the opinions or preferences of others are not necessarily better and that herding is a way of being accepted by the social group so as not to be rejected or punished (25). Of course, consumers do not herd for all products, and are less likely to herd for products that are used to express personal identity and highlight individual differences, such as clothing or music genres (26). Consumer behavior research has found that both stable personal characteristics and variable environmental factors drive consumers to be more or less herd-oriented. For example, consumer characteristic needs (27), volitional attributes (28) and cultural background (29) are all personal attributes of herding consumers that have been explored by scholars. Therefore, this paper proposes the hypothesis 4.

Second, we explain the hypotheses 5. The Health Belief Model suggests that individuals' perceptions of the likelihood and severity of illness are the driving force behind health behavior, and that personal and social characteristics “behind” the level of perception indirectly influence individual health behavior (30). Health food consumption is one of the many health behaviors that are indirectly influenced by health consciousness (31). As consumers become more health conscious, their need for health information increases. With objective and rational knowledge of food products, their choice behavior is less likely to follow objective rules and be blind (32). With regard to the factors influencing health awareness, a large number of scholars have verified that personal characteristics, family characteristics and health status play significant roles in health awareness and have explored regional differences in this relationship (33). Therefore, this paper proposes the hypothesis 5.

## 3. Research design

### 3.1. Data source and sample description

The data used in this paper are derived from household research conducted in June and July 2021 in four first-tier Chinese cities, including Beijing, Shanghai, Guangzhou and Shenzhen. The research used a combination of multi-stage stratified sampling and random sampling principles, with 3 to 4 randomly selected sample districts and counties in each city, 3 to 5 randomly selected natural villages or streets in each sample district and county, and 10 to 15 randomly interviewed consumers. A total of 30 districts and counties were involved in the survey, and a total of 1,713 questionnaires were distributed and collected, excluding samples with missing data, 1,658 valid questionnaires were distributed, with an effective rate of 96. 83%, which has good representativeness. The regional distribution of the sample is shown in Table 1.

**Table 1:** Regional distribution of the sample.

**City**	**District**	**Sample size**	**City**	**District**	**Sample size**
Beijing	Chaoyang District	114	Shanghai	Jing'an District	103
	Haidian District	113		Xuhui District	107
	Daxing District	102		Minhang District	109
	Xicheng District	105		Hongkou District	111
Guangzhou	Yuexiu District	113	Shenzhen	Futian District	69
	Hazhu District	105		Nanshan District	115
	Liwan District	101		Longhua District	86
	Tianhe District	112		Guangming District	93

In terms of organic food consumption, 1,479 respondents had consumed organic food (89.19%), and organic food accounted for 5.49% of food consumption on average; among them, 595 (35.91%) were consumers with an awakened sense of personal health while 350 (21.08%) were consumers with a herd mentality. In terms of individual characteristics, 816 (49.21%) of the respondents were male and 842 (50.79%) were female; the average age of the respondents was 32.77 years; and the average level of education was concentrated in the bachelor's degree (28.28%).

### 3.2. Variables and descriptive statistics

1. Dependent variable. The dependent variable in this paper is the share of consumers' organic food consumption in food consumption, which is a dimensionless continuous variable and requires no special treatment (25).

2. Core independent variables. The core independent variables chosen for this paper are “health consciousness” and “herd mentality” of consumers.

A consumer is considered to be health conscious if he or she (1) knows about organic food and the nutritional value of it and (2) insists on buying organic food even if others around him or her buy other similar products.

If (1) they have not heard of organic food or do not know about its nutritional value (2), they will buy organic food even when others around them buy organic food. They are considered to be a health conscious consumer and are classified as experimental group 2. Other consumers are classified as experimental group 0.

3. Control variables. In order to clarify the factors influencing consumers' beliefs about organic food consumption, individual characteristics, family characteristics, health status, quality of will, social trust and market education were selected as control variables. Four variables were selected for individual characteristics: gender, age, marital status, and education level. The differences in physiological characteristics and life experiences of different gender and age groups will result in different consumption beliefs (25). The marriage will also reshape individuals' perceptions and attitudes toward food and drink, and consumers with a high level of education will be more receptive to knowledge and have a healthier consumption philosophy. Three variables were selected for household characteristics (26): (1) Number of family members, number of dependents and household income. The number of people in the household influences the information received and the food consumption decision to a certain extent; (2) Families with a large number of dependents have to pay more attention to the health needs of the elderly and children (27); (3) The level of income determines the consumer class to which they belong, and there are differences in the consumption philosophy of different classes. The respondents' health status was characterized by two variables (28): (4) self-rated health and health concern, with those who were unwell and those who were concerned about their health caring more about their diet structure. The quality of will was characterized by adherence to a healthy routine (29), with those who were strong-willed being more likely to adhere to a good routine. In terms of social trust, those who trust in traceability and organic food are more likely to consume organic food and consume more of it. The market education selection focuses on the attention paid to organic food advertising, and advertising can effectively guide the public's consumption philosophy (30). The assignment notes and descriptive statistics of the above variables are shown in Table 2.

**Table 2:** Results of descriptive statistics for variables.

**Variable groups**	**Variables**	**Variable indicators**	**Sample size**	**Mean**	**Variance**	**Minimum**	**Maximum**
Effectiveness indicators	Organic food as a share of food consumption	%	1,658	5.494	9.477	0	80.6
Subgroup indicators	Derived from health awareness	0 = No; 1 = Yes	1,658	0.361	0.483	0	1
	Derived from herd behavior	0 = No; 1 = Yes	1,658	0.232	0.406	0	1
	Gender	0 = Female;1 = Male	1,658	0.492	0.500	0	1
	Age	—	1,658	32.771	18.530	16	90
Individual characteristics	Married or not	0 = No; 1 = Yes	1,658	0.704	0.457	0	1
	Education level	0 = No education; 1 = primary school; 2 = lower secondary school; 3 = upper secondary school; 4 = specialist; 5 = Bachelor; 6 = Master; 7 = Doctor	1,658	3.521	1.532	0	7
Family characteristics	Number of family members	—	1,658	3.713	1.478	1	15
	Number of dependents	—	1,658	1.153	1.238	0	6
	Household income	1 = less than 10,000; 2 = 1 to 20,000; 3 =2 0 to 30,000; 4 = 30 to 40,000; 5 = 40 to 50,000; 6 = 50,000 or more	1,658	4.491	1.732	1	6
Health status	Self-evaluation	0 = very poor; 1 = relatively poor; 2 = fair; 3 = better; 4 = very good	1,658	2.877	0.814	0	4
	Whether to be concerned about health	discrete values from 0 to 6, where 0 = no attention at all; 6 = extremely attentive; the higher the value, the stronger the degree of attention	1,658	3.287	1.547	0	6
Quality of will	Adherence to a healthy lifestyle	0 = poor; 1 = medium; 2 = good; 3 = excellent; 4 = very good	1,658	2.873	1.531	0	4
Social trust	Level of trust in traceability system	0 = Very distrustful; 1 = Relatively distrustful; 2 = No Feelings; 3 = more trusting; 4 = very trusting	1,658	2.677	0.802	0	4
	Whether to trust organic food	0 = Very distrustful; 1 = Relatively distrustful; 2 = No Feelings; 3 = more trusting; 4 = very trusting	1,658	2.614	0.867	0	4
Marketing education	Whether to see organic food related advertisements	0 = not available; 1 = less often; 2 = generally; 3 = more often; 4 = often	1,658	1.789	1.268	0	4

A counterfactual analysis framework for consumer psychology based on propensity score matching is proposed. The dummy variables were used to indicate whether the respondents belonged to the health consciousness, herd consumption or control group.

The specific analysis steps are as follows.

First, the covariates xi were selected, and exogenous influences on consumption beliefs and behavior, including individual characteristics, family characteristics, health status, volition, social trust, market education, and regional characteristics, were included in the model to meet the negligibility assumption.

Second, propensity scores were calculated. A Logit regression model was used to estimate the propensity score for respondent i's health awareness and the propensity score for herding behavior.

Third, the propensity scores were matched. (1) Selection of matching methods. There is some bias in the results of different matching methods for the same sample, but the methods themselves are not better or worse, and if similar or consistent findings can be obtained using multiple matching methods, the results are robust. Therefore, in order to ensure the reliability of the findings, five mainstream methods were selected for matching. The first method is k nearest neighbor matching, which finds the nearest individuals in different groups with different inclination scores, and sets k = 4 for 1-to-4 matching so as to minimize the mean squared error. The second method is Caliper matching, which matches different groups of individuals within absolute distance, and uses the default caliper range of 0.05 radius. The third method is bootstrap matching, which uses the default 1:1 proximity matching method and performs 500 times to obtain the mean value after sampling. (2) Test for balance. Once an accurate propensity score estimate is obtained, the matched experimental and control groups are tested for equilibrium of the covariate xi to ensure that the sample is equally distributed between the experimental and control groups.

Finally, mean treatment effects were calculated. This includes the average effect of the experimental group (ATT), the average effect of the control group (ATU) and the average effect of the full sample (ATE). The ATT for Experimental Group 1 represents the effect of personal health awareness on buckwheat consumption, the ATT for Experimental Group 2 represents the effect of herding behavior on buckwheat consumption, the ATU for the control group represents the mean value of the proportion of buckwheat consumed by the population when no action is taken, and the ATE for the full sample reflects the mean value of buckwheat consumption for a random sample. As this paper focuses on the contribution of health awareness and herding behavior to the consumption of mixed grains, it focuses on the comparison of the two experimental groups and therefore uses ATT for the analysis.

### 3.3. Analysis of the factors influencing consumer psychology

In order to achieve sample matching, this paper analyzed the key factors affecting consumption beliefs, and the results of logit estimation for personal health awareness and herding mentality are shown in Table 3. Pearson test and VIF test were conducted on the independent variables, and there was no multicollinearity problem.

**Table 3:** Logit regression results for consumer beliefs.

**Variable groups**	**Variables**	**Health awareness**		**Herd mentality**	
		**Regression coefficient**	**dy/dx**	**Regression coefficient**	**dy/dx**
Individual characteristics	Gender	−0.336^***^ (−2.790)	−0.067^***^ (−2.820)	−0.076 (−0.580)	−0.015 (−0.580)
	Age	0.029^***^ (5.430)	0.006^***^ (5.640)	0.002 (0.270)	0.000 (0.270)
	Married or not	0.242 (1.360)	0.048 (1.360)	0.035 (0.180)	0.007 (0.180)
	Education level	0.136^***^ (2.880)	0.027^***^ (2.910)	−0.149^***^ (−2.940)	−0.030^***^ (−2.980)
Family characteristics	Number of family members	0.216^***^ (2.610)	0.025^***^ (2.640)	0.056 (1.040)	0.011 (1.040)
	Number of dependents	−0.195^***^ (−3.320)	−0.039^***^ (−3.360)	−0.119^*^ (−1.849)	−0.019^*^ (−1.859)
	Household income	−0.039 (−1.428)	−0.009 (−1.338)	0.019 (0.498)	0.003 (0.498)
Health status	Self–evaluation	0.225^***^ (2.998)	0.044^***^ (3.029)	0.098 (1.119)	0.019 (1.119)
	Whether to be concerned about health	0.004 (0.149)	0.002 (0.148)	0.073^*^ (1.668)	0.016^*^ (1.677)
Quality of will	Adherence to a healthy lifestyle	0.369^***^ (4.509)	0.074^***^ (4.619)	0.355^***^ (3.969)	0.071^***^ (4.059)
Social trust	Level of trust in traceability system	0.203^**^ (2.169)	0.039^**^ (2.189)	0.091 (0.869)	0.018 (0.879)
	Whether to trust organic food	0.139 (1.519)	0.026 (1.519)	−0.096 (−1.009)	−0.019 (−1.019)
Marketing education	Whether to see organic food related advertisements	0.217^***^ (4.510)	0.043^***^ (4.462)	0.248^***^ (4.593)	0.051^***^ (4.839)
Model parameters	Constants	−5.443^***^ (−10.500)		−2.375^***^ (−4.350)	
	*Pseudo R* ^2^	0.1543		0.0618	
	*LR chi*^2^ (15)	317.09		96.13	
	*N*	1,489		1,218	

^*^, ^**^, and ^***^Indicate that the estimates are significant at the 10, 5, and 1% levels respectively, with the t-statistic in parentheses.

Table 3 shows that differentiated individual characteristics, family characteristics, health status, quality of will, social trust and market education are important factors in consumer health awareness and herd mentality. There is no significant difference in herd consumption; education is a key factor in health awareness and herd mentality, with more educated groups being more health conscious and less likely to herd. Household characteristics show that consumers from large families are more health conscious, but those from households with a large number of dependents, such as the elderly and young children, are less health conscious.

The reasons for this are that: On the one hand, it is the access to information while having a large family helps to increase knowledge of health information, and on the other hand, it is the access to time constraints while consumers with large families have a heavier household burden and less time to take care of their dietary health (31). In terms of health status, consumers who feel good about themselves are more health conscious, but the more they care about their health, the more likely they are to follow the trend of consumption. In terms of quality of will and social trust, strongwilled consumers are more pronounced in both health awareness and herd consumption; those who have a high level of trust in the food safety traceability system have a significantly higher proportion of health awareness awakening, reflecting that those who trust the food safety system are more rational consumers (32). In terms of market education, the more advertising education received, the more health conscious consumers are, but also the more likely they are to follow the herd. In summary, Hypothesis 4 and Hypothesis 5 were tested.

## 4. Measuring the effect of health consciousness and herd mentality on organic food consumption

### 4.1. Analysis of PSM matching results

In this paper, the fitted values of the conditional probabilities pi for respondent i's personal health awareness and herd mentality, i.e., the propensity score, were calculated based on the estimation results of the consumption belief equation. The maximum loss of samples in the matching method chosen for the study was 6, still retaining 1,652 matched samples with good matching results (see Table 4).

**Table 4:** PSM matching results.

**Projects**	**Matching samples**	**Unmatched samples**	**Total**
Control group	597	0	597
Experimental group 1 (health awareness)	687	6	693
Experimental group 2 (submissive behavior)	368	0	368
Total	1,652	6	1,658

### 4.2. Equilibrium tests

In order to ensure the reliability of the matching results and to satisfy that there were no significant differences in the influencing factors except for the differences in organic food consumption, a balance test of the covariates was required (33). The results are shown in Tables 5, 6. After sample matching, the standard deviation of the explanatory variables for health consciousness decreased from 29.3 to 2.7–4.0%; the overall bias was significantly reduced and all were within the 10% good standard line of the balance test; the *Quasi*−*R*^2^ decreased from 0.138 before matching to 0.003–0.008 after matching; and the LR statistic decreased from 319.37 to 8.27–18.29. The standard deviation of the explanatory variables for follower behavior decreased from 17.2 to 1.3–3.2%; the *Quasi*−*R*^2^ decreased from 0.059 before matching to 0.003–0.007 after matching; and the LR statistic decreased from 98.93 to 3.17-6.17. In terms of the deviation of each explanatory variable in the two experimental groups from the control group, except for the health consciousness education and market education of the awakened group, there were no significant differences. Together, this suggests that the propensity score matching method significantly reduced the differences in the distribution of explanatory variables between the two experimental groups and the control group, and largely eliminated the bias caused by sample selection.

**Table 5:** Results of balance tests before and after matching of health awareness explanatory variables.

**Matching method**	**Quasi−R^2^**	**LR statistic**	**Standardized deviation (%)**
Before matching	0.138	319.37	29.3
K Nearest neighbor matching	0.008	18.29	4.0
Caliper match	0.005	9.32	2.7
Within caliper k nearest neighbor matching	0.003	8.27	3.1
Kernel matching	0.006	12.37	4.0

The Bootstrap matching method was performed 500 times and the results of the parameters are not shown due to space constraints.

**Table 6:** Results of balance tests before and after matching of followership behavior explanatory variables.

**Matching method**	**Quasi−R^2^**	**LR statistic**	**Standardized deviation (%)**
Before matching	0.059	98.93	17.2
K Nearest neighbor matching	0.007	4.88	3.2
Caliper match	0.003	3.17	1.3
Within caliper k nearest neighbor matching	0.005	6.17	3.1
Kernel matching	0.003	3.3	2.8

The Bootstrap matching method was performed 500 times and the results of the parameters are not shown due to space constraints.

### 4.3. The effect of health consciousness and herd mentality on organic food consumption

The propensity score matching model estimated a net effect of 3.259 for the effect of personal health awareness on organic food consumption, indicating that after accounting for the bias in consumer health awareness, concern for dietary health contributed to a significant increase of 3.259% in the proportion of consumers consuming organic food. This confirms hypothesis 1.

The net effect of herding behavior on consumers' organic food consumption was 2.152, indicating that, after taking into account the herding mentality bias of consumers, following the herd contributed to a significant increase of 2.152% in the proportion of consumers consuming organic food; so the hypothesis 2 was verified. The net effect of health awareness was significantly higher than that of herding behavior, reflecting the fact that health awareness is the main driver of organic food consumption in China, but herding also plays an amplifying role.

It is worth noting that the increase in demand driven by health consciousness is a rational decision driven by consumers' objective perception of dietary health, and that demand for organic food driven by this driver is relatively stable, while organic food consumption driven by herd mentality tends to fluctuate according to the shift in consumption hotspots (33).

The degree of influence of health awareness and herd mentality varies considerably between different types of consumers, depending on their socio-cultural, educational and life experiences (16). In this paper, we will further explore the cohort differences among different types of consumers, based on Bootstrap method with 500 samples, and finally estimate the cohort difference comparison results of the effect of individual health awareness and herding behavior on organic food consumption as shown in Table 7.

**Table 7:** Results of heterogeneous group differences.

**Heterogeneous variables**	**Classification criteria**	**Health awareness**		**Herding behavior**	
		**Mean treatment effect**	**Standard deviation**	**Mean treatment effect**	**Standard deviation**
Gender	Male	3.933^***^	0.849	2.876^***^	1.008
	Female	2.989^***^	0.838	1.948^***^	0.698
Age	35 years old and below	1.897^**^	0.594	0.641	0.714
	35 to 45 years old (inclusive)	2.919^**^	1.498	1.593	2.113
	45 to 55 years old (inclusive)	4.173^***^	1.367	4.279^**^	1.689
	55 years old and above	2.498^**^	1.189	3.998^***^	1.453
Education level	Primary and below	1.608	2.107	5.583^**^	2.196
	Lower secondary	2.389^*^	1.492	3.258^**^	1.387
	High School	3.914^***^	0.979	1.509^*^	0.939
	University and above	1.814^***^	0.734	1.953^*^	1.009

^*^, ^**^, and ^***^ indicate that the estimates are significant at the 10%, 5%, and 1% levels respectively.

First, the net effect of organic food consumption, both in terms of health consciousness and herding behavior, is higher for men than for women. This reflects the fact that men have a relatively homogeneous diet, preferring to consume large quantities of a few foods, while women have a more diverse food choice, consuming more varieties but smaller quantities. Second, in terms of age structure, the net effect of health awareness on organic food consumption tends to increase and then decrease with the accumulation of life experience and the objective needs of the body. The net effect of health awareness on organic food consumption is greatest among consumers aged 45–55. Again, the net effect of health consciousness on organic food consumption tends to increase and then decrease with education level. There was no significant difference between the primary school and below educated group and the control group, where health consciousness awareness did not affect their organic food consumption; the highest effect of health consciousness on organic food consumption was observed for the high school educated group; while the significant effect of health consciousness on organic food consumption was lower for the university and above educated group. The highly educated group is more diversity-oriented and moderately increases organic food consumption (34). Finally, the net effect of herding behavior on organic food consumption tends to decrease with increasing education, indicating that the level of education influences consumers' learning and vision to a certain extent, and the net effect of herding behavior decreases with higher education levels. Hypothesis 3 was verified.

## 5. Conclusions and recommendations

Based on data from 1,658 respondents in four first-tier cities in China, this paper explains whether consumers' organic food consumption is motivated by health awareness or herding behavior. This paper uses a logit model to explore the key triggers of health awareness and herding behavior, and uses the propensity score matching (PSM) method to measure the effects of health awareness and herding behavior on organic food consumption. The effects of health awareness and herding behavior on organic food consumption were measured using the Propensity Score Matching (PSM) method and were compared across age and education groups. The study shows that individual characteristics, family characteristics, health status, volition, social trust and market education significantly influenced consumers' health awareness and herding behavior; the main driver of organic food consumption was health awareness, but herding also played a role in amplifying the effect.

Based on the above findings, this paper proposes the following recommendations to further optimize consumers' dietary structure and promote the quality development of the organic food industry.

1. We should give full play to the driving role of the awakening of health awareness in the growth of coarse grain consumption, strengthen dietary health education, and advocate healthy consumption and rational consumption. As education is the foundation of the country and the key to improving the quality of the people, we can promote healthy diet and improve the “online + offline” official publicity system of food culture education, so that the improvement effect of health awareness awakening can be fully released.

2. We should face up to the herd mentality to the grain industry. The role of promotion is to make proper use of herd psychology to increase market demand while taking preventive measures. The increased demand for herd consumption is easy to fade with the transfer of consumption hotspots, resulting in large fluctuations in market prices and affecting the steady development of the grain industry. Therefore, it is necessary for the government to guide consumption trends and prevent the market impact caused by the frequent switching of consumption hotspots between different foods. The government should regulate product advertising, punish false and exaggerated publicity, and avoid price fluctuations caused by false propaganda that is caused by follower consumption.

3. We should focus on the information needs of special groups and do a good job of point-to-point assistance. For groups who have no time to take care of healthy diets due to heavy family burdens and high support pressure, relevant government departments can push healthy and reasonable dietary structures for them through service platforms such as text messages. For middle-aged and elderly groups who are easy to consume herdly, we should actively carry out the activity of “guiding and protecting the rights of elderly consumer subjects,” and improve the dietary guidance of middle-aged and elderly consumer groups.

## Data availability statement

The original contributions presented in the study are included in the article/supplementary material, further inquiries can be directed to the corresponding author.

## Ethics statement

The studies involving human participants were reviewed and approved by Ethics Committee of Shenzhen Polytechnic, China. Written informed consent to participate in this study was provided by the participants' legal guardian/next of kin.

## Author contributions

First draft writing: JW. Review writing: YX and TL. All authors contributed to the article and approved the submitted version.

## Footnotes

^1^ https://knowledge4policy.ec.europa.eu/publication/world-organic-agriculture-statistics-emerging-trends-2022_en
